# Using a Nominal Group Technique to Develop a Science Communication Curriculum for Health Professionals and Clinical Researchers

**DOI:** 10.1007/s13187-023-02282-z

**Published:** 2023-03-17

**Authors:** Meredith Elana Giuliani, Tina Papadakos, Catherine Coolens, Jose Fregnani, Philipp Gebhardt, Anet Julius, C. S. Pramesh, Naa Kwarley Quartey, Janet Papadakos

**Affiliations:** 1 Princess Margaret Cancer Centre, University Health Network, Toronto, Canada; 2 A.C Camargo Cancer Centre, Sao Paulo, Brazil; 3 German Cancer Research Center (Deutsches Krebsforschungszentrum), Heidelberg, Germany; 4 Tata Memorial Cancer Centre, Tata Memorial Hospital, Mumbai, India

**Keywords:** Nominal group technique, Curriculum, Science communication, Health professions education, Knowledge translation, Global, Cancer

## Abstract

Effective science communication is fundamental to closing the gap from research and innovation to clinical implementation. Existing paradigms of science communication are often challenged by a lack of skill and engagement, particularly from those who progress the science. Currently, a standardized curriculum on science communication, with global applicability, does not exist. The purpose of this project is to address the gap in training by health professionals and clinical researchers through the development of a globally relevant curriculum for science communication. The nominal group technique (NGT) was used whereby a convenience sample of eleven science communication experts from across the globe generated, discussed, and arrived at a consensus on topics that should be included in a standardized science communication curriculum. Experts represented diverse backgrounds within the health sciences. Due to the COVID-19 pandemic and geographical constraints, the NGT was conducted virtually. The consensus-building methodology allowed for each expert to equally present ideas and collaborate with one another to create a robust and comprehensive curriculum for effective science communication. Expert panelists reached a consensus on 10 essential components of a standardized global science communication curriculum. Following the refinement of the curriculum topic areas, a virtual meeting with project co-investigators was held to review the topics and discuss relevance, applicability, and appeal to the local contexts. A standardized science communication curriculum is needed for health professionals and clinical researchers. The NGT achieved expert consensus on the core topics. The next steps are to develop the course ensuring optimal participation from learners across the globe.

Globally there is a profound inequality in access to cancer care and these gaps are projected to increase in the next decade. The causes of these gaps are multifactorial, including health human resource shortages, lack of access to cancer medicines, radiotherapy, diagnostic tools, and health systems infrastructure gaps. The know-do gap [1, 2], or the time it takes for new knowledge to diffuse into clinical practice, is very slow across the globe, with some quoting this interval to be 17 years [3]. One avenue to reduce the know-do gap is to further improve the skills of clinicians and researchers in the domain of science communication. Leveraging the power of science communication and improving personal skills training in this area can support and enable stakeholders, including scientists, clinicians, administrators, policymakers/politicians, and the general public, to understand and act on scientific advances [2]. Indeed, many implementation scientists have noted that the knowledge gap is caused, in part, by a translation deficit [2]. Translation requires a constellation of science communication skills and despite the importance of science communication, important curricular frameworks, like the CanMEDS 2015 framework, developed in Canada and implemented in dozens of countries, do not include a detailed curriculum for effective science communication [4].

## Problem

**Fig. 1:**
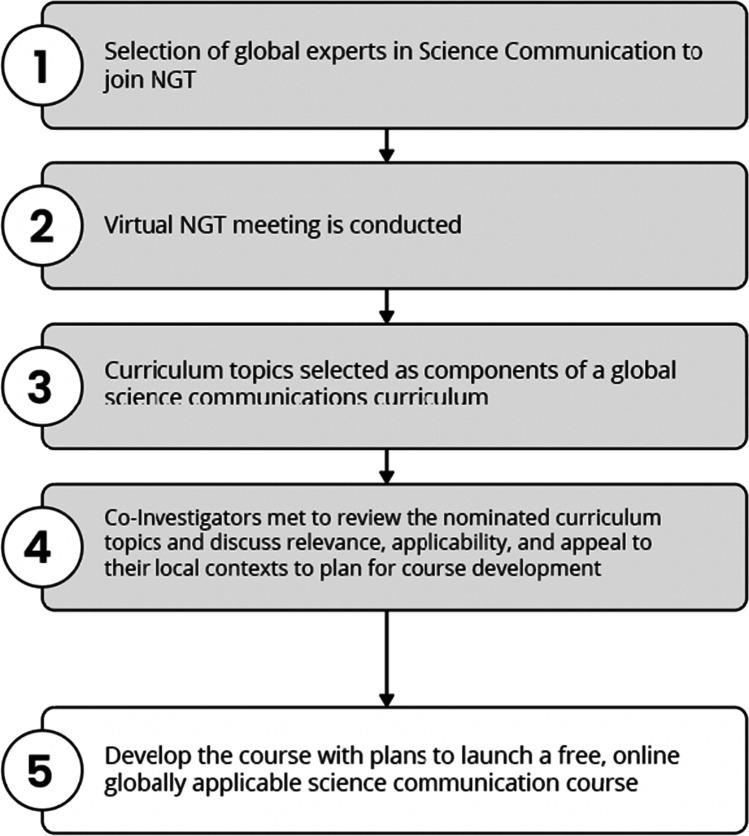
Steps undertaken to develop the curriculum outline

The current paradigms of science communication are often challenged by a lack of skill and/or engagement from those progressing in science. One cause for this is certainly that science communication modules for a long time have not been part of the educational programs for researchers and clinicians. However, we slowly see increasing interest and progress in certain countries and specific institutions where these elements are being introduced to complement the individual training portfolios for these target groups. Another reason might be that traditional venues for science communication steeped in Western values and priorities fail to address the global cancer control agenda, and in addition, there is a lack of attention to digital and social mechanisms for information diffusion. To realize success on a global scale, one must also address issues of neo-colonialism, which permeate medical science, education, and clinical practice [5]. Curriculum development efforts should therefore involve the engagement of stakeholders representing diverse perspectives on global science, health systems policy, and clinical practice. Currently, Western priorities and science are at the core of many international medical training programs [6]. The power differentials in the Global North versus the Global South have been proposed to perpetuate Western or Euro-American beliefs and priorities at the expense of local knowledge [7]. It is clear that there is a need globally for deliberate training in science communication [8]. Without intentional efforts, rooted in the concepts of health, media, and cultural literacy, it is unlikely that the research to policy to practice gaps will close [1]. In fact, as evidenced during the COVID-19 pandemic, these concerns may further widen disparities in care [9]. These efforts should leverage global expertise in the area of science communication. The purpose of this work was to address the gap in medical and research training by developing a globally relevant curriculum for science communication in the health professions. It should be noted that the project co-investigators work in the oncology setting; however, the process for curriculum development was not directly tied to cancer. From the outset of this work, the co-investigators had planned to develop case studies from varying oncology contexts to demonstrate different approaches taken to communicate science as described in this manuscript. The co-investigators undertook a 5-step process to endeavor to develop a globally relevant science communication curriculum. The process is described in detail below, and Fig. 1. provides an overview of the steps.

## Approach

### Nominal Group Technique Background

This curriculum development work employed a qualitative method, the nominal group technique (NGT). Since its development in 1960, the NGT has been used in education and health, among other fields, as a consensus development method [10–12]. It can be used as an alternative to the Delphi technique and focus groups [13]. The NGT addresses some of the limitations of Delphi in curriculum development by weighing all views equally and allowing all group members to express their opinions and allowing for a more structured approach than focus groups [14]. The main goal of the NGT is to resolve an issue by generating information and establishing priorities through group discussion [12]. In the nominal group technique, 6–12 participants are invited to a single 2-h discussion session led by a moderator [13]. Both participants and the facilitator are required to be subject matter experts of the topic under discussion [12]. NGT participants are not required to have any preparation prior to the NGT session.

**Fig. 2:**
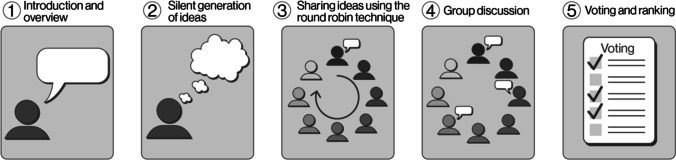
Steps to perform the nominal group technique (NGT)

The NGT is composed of five consecutive steps (see Fig. 2) [11, 13]. The first step is the introduction of participants and the moderator as well as an explanation of the purpose and the steps of the meeting [11]. The second step is the silent generation of ideas, where participants are given time to answer specific questions asked by the moderator and then are instructed to write down their ideas and not to consult or discuss their answers with the other participants [12]. The third step consists of participants sharing ideas using cycles of the round-robin technique, where each participant shares one thought in turn and the cycle continues until everyone has presented all of their ideas [11]. During this step, the moderator needs to remind all participants that there is no debate or interruptions until everyone has contributed. All the ideas shared during this step need to be recorded verbatim to be presented later to the group [11]. Step 4 is a group discussion, and participants are asked to request verbal explanations and details about any unclear ideas shared by others. In this step, the moderator needs to manage the discussion in a way that allows participants to contribute equally, without letting one person dominate the discussion while engaging the quieter participants. It is important to remind participants to avoid judgment and criticism during this time so the process can remain value neutral [11]. The fifth and last step is voting and ranking. Participants are instructed to prioritize their ideas in relation to the original research questions and to negotiate with other participants about the top priorities [11]. After this step, responses are readily available for participants, and the session can be concluded having achieved a specific outcome [12].

### Conducting the NGT

This work was conducted in June 2021, and due to the COVID-19 pandemic and geographical constraints, the NGT was conducted virtually. Science communication experts were identified through convenience informed by snowball sampling through the co-investigators who were familiar with the expert’s work through personal networks. In order to obtain a sample of participants with global representation, seven geographical regions were targeted: Asia, Africa, North America, South America, Antarctica, Europe, and Australia. The co-investigators’ home countries included Canada, Brazil, Germany, and India, and therefore, several participants were invited from these countries within the respective seven regions. The Internet was searched to help identify science communications experts from regions where the co-investigators did not have contacts. For example, the co-investigators were not familiar with science communication experts from Antarctica and thus used the Internet to search for and identify individuals with suitable expertise. Science communication experts had diverse backgrounds within the health sciences such as education, journalism, biomedical research, and medicine. The ranges of expertise in the science communication field varied from advanced to moderate expertise. For example, advanced participants included professors and directors of university graduate programs in science communication, and participants with moderate expertise included clinician-scientists with a passion for communication and expertise in their local setting (e.g., academic teaching hospital). A total of thirteen science communication experts from six of the seven regions, representing ten countries (Australia, Brazil, Canada, Chile, China, Germany, India, Rwanda, Singapore, and Zambia), were invited via email to participate in the NGT session and eleven accepted the invitation. Unfortunately, participants from Singapore and Zambia were unable to attend.

The NGT was conducted virtually over 3 h, and consent was obtained from participants to record the session. After the session, participants were provided with an honorarium (CAD $150). Following the NGT protocol steps, the session started with the moderator welcoming participants and inviting them to introduce themselves. The moderator is an education scientist and experienced facilitator with 15 years of experience in teaching and training. Once introductions were completed, the moderator gave a brief introduction on the nominal group technique steps, the rules that had to be followed, and the time allotted for each step. The moderator presented the aim of the NGT session: to develop curriculum topics for a science communication training course. The second step was the silent generation of ideas. In this step, participants were presented with one question: “What topics should be covered in a science communication course for health professionals and clinical researchers”? Participants were asked to consider science communication in the context of scholarship, public communication, policymakers, patients and families, scientist-clinicians, decision-makers, advocacy, and philanthropy. No definition of science communication was provided as NGT participants had expertise in the subject, and project investigators wished to avoid confining the breadth of ideas. There were no constraints on the number of curriculum topics. The moderator requested participants to turn off their cameras and microphones and to write down all the answers they could think of, and they were instructed to refrain from communicating with each other. Experts were given 10 min to complete this step.

Step 3 consisted of participants sharing their ideas one by one in a multiple rounds (round-robin) format. If the expert did not have any ideas to share, they could pass the opportunity to share to the next expert. A research analyst and a research associate transcribed each idea presented by the experts. Approximately ten rounds of idea sharing occurred until experts had no further ideas to share. The intention of this step was to allow participants to share the ideas they came up with during the silent generation of ideas and by listening to the other experts share their ideas and trigger the conception of more ideas. This step lasted 90 min. After this step, participants were given a 15-min break period while the research analyst and the session moderator organized the information obtained from the idea sharing in a PowerPoint slide, so the experts were able to visualize the curriculum topics and conduct the next step.

Once participants returned from break, they were presented with the slide and the moderator asked participants if any ideas were unclear or required any clarification and further discussion. The experts spent 20 min discussing and clarifying ideas while the facilitator moderated the discussion. When the idea discussion was completed, the group proceeded to the final step of voting and ranking ideas. The research assistant sent a link to Slido, an online platform for voting and ranking, where the curriculum topics were shown in no particular order. Participants were asked to rank their choice of ideas from most important to least important in relation to the original question. This step lasted 15 min and concluded once all participants had submitted their votes.

## Outcomes

Sixty-five curriculum topics were generated during the round-robin. Several curriculum topics overlapped, and the research analyst and moderator thematically organized and condensed topics together into thirteen main curriculum topics with thirty-six sub-topics. The main curriculum topics were as follows:

1. Knowledge user engagement (e.g., how to conduct needs assessments, market research)
2. Basic psychology (e.g., cognitive bias, memory, and cognitive overload)
3. Education science (e.g., adult learning, meaning making)
4. Social determinants of health (e.g., health literacy and factors that impact access to information)
5. The art of persuasion and rhetoric (e.g., figures of speech (metaphors) and types of appeal in terms of pathos (emotions, values that are intrinsic to meaning), ethos (moral standing of a person), logos (thoughtful reflection with data/facts))
6. Communication strategies (e.g., plain language, dealing with misinformation, disinformation, and controversy)
7. Equity diversity inclusivity and accessibility (EDIA) (e.g., discourse analysis, unconscious bias, community engagement)
8. Communicating with the media (e.g., appreciating the needs of the journalist, how to write an elevator pitch)
9. Social media (e.g., best strategies to maximize impact)
10. Empathy and compassion in science communication (e.g., appreciating the needs of different audiences and the impact of communication)
11. Ethics in science communication (e.g., advocacy impactful change)
12. Design principles (e.g., modality selection, design tools)
13. Uses of science communication (e.g., patient/public education, career progression, and promotion, international collaboration)

Nominated curriculum topics were ranked by participants, and consensus was achieved with ten curriculum topics being selected as core components of a global science communications curriculum:

1. Engaging knowledge users
2. Communication strategies
3. Basics of psychology
4. Uses of science communication
5. Social determinants of health
6. Equity diversity inclusivity and accessibility
7. Ethics of science communication
8. Art of persuasion and rhetoric
9. Empathy and compassion in science communication
10. Education science

Following the NGT session, the moderator and a co-investigator who is an instructional designer, both of whom have expertise in science communication, further refined the curriculum topics in terms of flow, redundancy, and expansion of some topic areas. The revised curriculum included ten major topics:

1. Introduction to science communication
2. Knowledge user engagement
3. Education science and behavioral psychology basics
4. Art of persuasion and rhetoric
5. Health literate communication strategies
6. Equity diversity inclusivity and accessibility
7. Media engagement
8. Strategic communication
9. Science visualization and presentation skills
10. Dissemination of research evidence

The next step in the curriculum development process was a meeting of project co-investigators (with representation from Brazil, Canada, India, and Germany) to review the nominated curriculum topics and discuss, to the best of their ability, the relevance, applicability, and appeal to their local contexts to plan for course development. The meeting was held virtually, 1 month following the NGT session. It was structured using a framework for curriculum planning called the “3Ws and an H” [15]. Using this framework, co-investigators were asked to consider the who-why-what and how of the course: Who is the target audience(s) for the course? Why would each target group want to take the course? What topics would learners like to learn about most? and How should the course be delivered to optimize participation?

Through this meeting, the project investigators agreed that the course should be targeted toward clinician-scientists, healthcare professionals, and health researchers. The project investigators cited motivations for these individuals to participate in the course as promoting their research, writing applications for funding including writing lay summaries, sharing their research with non-experts, and communicating complex information to patients, family, and the public. When asked which curriculum topics would be most useful to learners, project investigators differed in the order of importance of subjects but agreed that all ten of the refined topics would appeal to learners in each of their country contexts. When discussing how the course should be delivered to optimize participation from learners across the globe, project investigators shared advantages and disadvantages of synchronous and asynchronous courses. The advantages of a synchronous course delivery included real-time feedback from course instructors and opportunities for networking among learners. Disadvantages of synchronous course delivery included temporal limitations given divergent time zones and limitations of transmission speeds (bandwidth) in rural settings. These important disadvantages held force to sway all project investigators to agree to develop a curriculum for asynchronous delivery.

Effective training in science communications is becoming more important as health professionals and researchers recognize the importance of closing the know-do gap. While the NGT process has successfully been used in curricula development [16–18], to our knowledge, our work demonstrates the first time a group of global experts from several countries have come together to establish consensus on the topics that should be included in a global science communications curriculum. The NGT process allowed for equal representation from all global experts and also allowed the group to discuss and provide feedback immediately. Conducting the NGT virtually removed potential barriers to ensuring global panelists would be able to participate in establishing a framework for building a science communication curriculum that has a global reach.

The authors invited science communication experts who participated in the NGT to aid in the development of a new global science communications course specifically targeted to health professions and clinical researchers. The topics identified during the NGT did not differ significantly from the extant science communication literature. The major difference between the published science communication literature and the topics that emerged from the NGT is context and the way that the various topics are appreciated within different global contexts. The science communication course that we are developing as a result of this work includes case studies from across the globe that are meant to share diverse perspectives on common science communication topics.

In terms of how the topics identified through the NGT compare to the CanMEDS framework, the topics differ fairly significantly. The CanMEDS role of “communicator” is the role within the framework where we would most expect to see elements of science communication competency. The communicator role has 24 key concepts associated with it and when we examine the ten topics identified for a priority through the NGT, there is only minor overlap. Two topics from the NGT specifically overlap with CanMEDS key concepts. The NGT topic, “Health literate communication strategies” overlaps with three key concepts in the CanMED communicator role, Active listening, Effective oral and written communication, and Patient-centered approach to communication. The NGT topic, “Equity diversity inclusivity and accessibility,” has some overlap with two of the CanMEDS key concepts, Respect for diversity and Trust in physician/patient relationships.

## Next Steps

Currently, several experts are working collaboratively to develop the course with plans to launch a free, online globally applicable science communication course in the fall of 2023. Developing the curriculum with experts from several countries may support the creation of learning experiences that are applicable to learners living in different contexts in various parts of the world, in contrast to content developed locally by faculty living in the same region as learners.

In addition, the curriculum developers asked NGT participants to identify local science communicators that would be invited to participate in a single interview to support the development of case studies. The case studies are meant to provide a sampling of different approaches to communicating science based on available media channels and cultures. Science communicators from Chile, Colombia, Japan, the Philippines, and Kenya have been invited to interview and they are scheduled to take place in the near future. Neo-colonialism pervades global discourses where the Global North is often narrating stories and setting standards for the Global South. An epistemological change is emerging where the Global South is valued for novel approaches to solving complex problems, and teaching and learning are more reciprocal. Our effort to include case studies demonstrating novel approaches from the Global South is one way that the co-investigators are endeavoring to address this challenge.

The opportunity to create a course with broad applicability is increasingly significant for many reasons including the impact of free online courses in reducing barriers associated with affordability and access, and the growing appreciation for the value of community engagement and tailored implementation of evidence for best outcomes. This project has yielded a basic science communication curricular framework for health professionals and clinical researchers with global applicability. Science communication skills are fundamental to closing the research and innovation to clinical implementation gap. The authors support that all health professionals and clinical researchers require training in this area. Currently, communication skills outlined in training frameworks such as CanMEDS [4] focus on communication skills with patients and families only and in the scholarly domain, research skills are present but the skills specific to science communication are not clearly articulated. Consideration must be given to expanding current training programs to include science communication to course curricula to better equip health professionals and clinical researchers to communicate their science to support understanding and uptake.
